# Prediabetes and the incidence of Parkinson’s disease: A meta-analysis

**DOI:** 10.17305/bb.2023.10035

**Published:** 2024-08-01

**Authors:** Xiaojie Jin, Yi Lu, Zhongying Gong, Weihui Huang, Zhiyun Wang

**Affiliations:** 1 Department of Neurology, Tianjin First Central Hospital, School of Medicine, Nankai University, Tianjin, China

**Keywords:** Prediabetes (PreD), hyperglycemia, Parkinson’s disease (PD), incidence, meta-analysis

## Abstract

Diabetes has been associated with an elevated risk of Parkinson’s disease (PD), yet the relationship between prediabetes (PreD) and the incidence of PD in the adult population remains unclear. Therefore, a systematic review and meta-analysis were conducted to evaluate if PreD is also associated with a higher risk of PD. We conducted comprehensive searches of the PubMed, Embase, and Web of Science databases to identify relevant observational studies with longitudinal follow-up. The random-effect model was employed to synthesize the data, mitigating the potential impact of study heterogeneity on the outcomes. Our analysis incorporated seven datasets from five cohort studies, encompassing 18,170,592 adult participants without a PD diagnosis at baseline. Among them, 2,432,148 (13.3%) had PreD. During the follow-up, a total of 46,682 patients were diagnosed with PD. The pooled results indicated that PreD was associated with an increased incidence of PD (risk ratio [RR]: 1.09, 95% confidence interval [CI] 1.02–1.16; *P* ═ 0.02; *I*^2^ ═ 52%), after adjusting for potential confounding factors, such as age, sex, body mass index (BMI), and smoking. Subsequent pilot subgroup analyses suggested that the association between PreD and PD might not be significantly influenced by the country of the study, its design, the age or sex of the participants, the definition of PreD, or the quality scores of the study (*P* for subgroup difference all > 0.05). In conclusion, the adult population with PreD may have a mildly increased risk of developing PD compared to those with normoglycemia.

## Introduction

Parkinson’s disease (PD), the second most prevalent neurodegenerative disorder, affects approximately 1% of individuals over 50 years of age [1, 2]. PD patients experience a gradual loss of dopaminergic neurons in the substantia nigra, leading to cardinal motor symptoms, such as tremor, rigidity, and bradykinesia, which significantly diminish their quality of life [3, 4]. Despite ongoing research, the precise mechanisms underlying PD remain incompletely understood, making it crucial to elucidate risk factors for PD in the general population [5].

Diabetes, a group of metabolic disorders, arises from either an absolute or relative insulin secretion insufficiency and/or dysfunction in insulin utilization and is primarily characterized by hyperglycemia. The pathophysiology of diabetes involves the deterioration of pancreatic islet beta cell function and the development of insulin resistance, which underpins its pathogenesis [6, 7]. Over recent decades, the prevalence of diabetes has reached epidemic proportions [8, 9], with global statistics from 2019 indicating that over 463 million individuals are affected [10]. Clinically, diabetes is known for complications impacting both microvascular and macrovascular systems [11, 12]. Accumulating evidence has also established a connection between diabetes and the pathogenesis and progression of neurodegenerative diseases, including PD [13, 14]. In recent decades, research interest has increased in prediabetes (PreD), a mildly glycemic disorder that exists between normoglycemia and diabetes [15, 16]. Clinically, PreD could be defined by the presence of impaired fasting glucose (IFG), impaired glucose tolerance (IGT), and mildly elevated glycated hemoglobin (HbA1c) [17, 18]. Similar to diabetes, PreD places individuals at a heightened risk for various vascular complications [19, 20]. Some preclinical studies have suggested that persistent hyperglycemia could contribute to neuronal dysfunction and death, potentially inducing PD, raising the hypothesis that hyperglycemia may be involved in the pathogenesis of PD [21]. However, previous studies examining the association between PreD and the risk of PD have yielded inconsistent findings [22–26]. Consequently, a systematic review and meta-analysis were conducted to evaluate whether PreD is associated with an increased risk of PD.

## Materials and methods

The study adhered to the guidelines set forth by the Preferred Reporting Items for Systematic Reviews and Meta-Analyses (PRISMA) statement [27, 28] and the Cochrane Handbook of Systematic Reviews of Interventions [28] throughout all stages of planning, conducting, and reporting.

**Figure 1.**
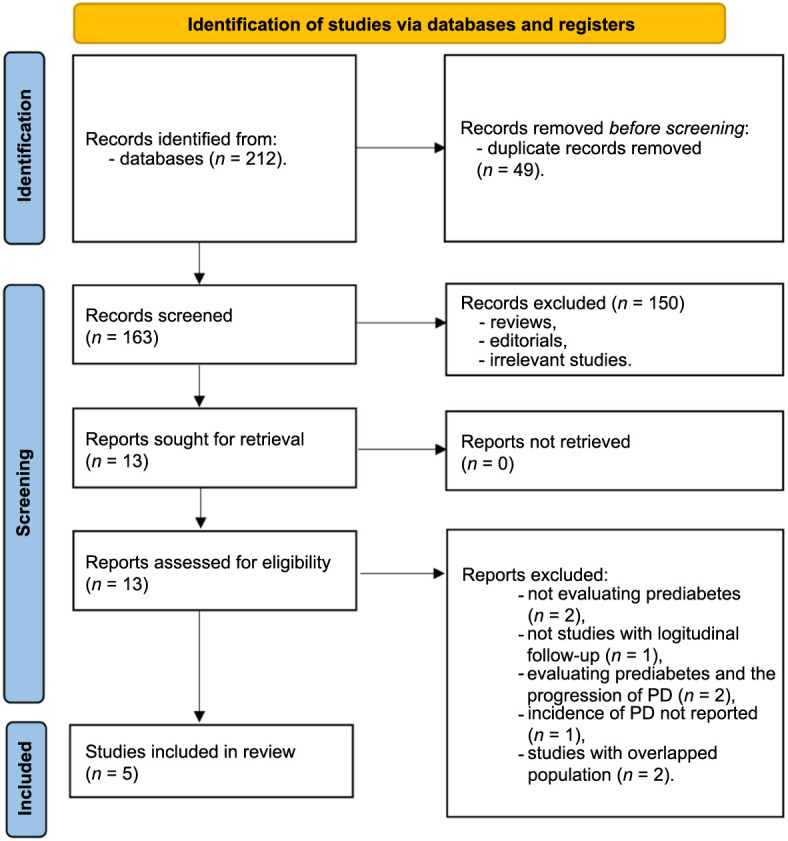
**Flowchart of database search and study inclusion process.** PD: Parkinson’s disease.

### Inclusion and exclusion criteria of studies

In formulating the inclusion criteria for the study, the Population, Intervention, Comparison, Outcomes, and Study (PICOS) design guidelines were meticulously followed, ensuring alignment with the objectives of the meta-analysis. The participants (P) included in the study were adults who did not have a confirmed diagnosis of PD at baseline. The exposure (I) group consisted of participants diagnosed with PreD, as defined by the methods and criteria used in the studies included in the analysis. The control (C) group comprised participants with normoglycemia. The outcomes (O) of interest were the reported risk ratios (RR) for the incidence of PD, comparing patients with PreD to those with normoglycemia, with PD diagnosis methods consistent with those applied in the original studies. Regarding the study design (S), the focus was on observational studies with longitudinal follow-up, which encompassed cohort studies, nested case-control studies, and post-hoc analyses of clinical trials.

**Table 1:** Characteristics of the included studies

**Study**	**Country**	**Design**	**Population**	**Sample size**	**Mean age (years)**	**Men (%)**	**Diagnostic criteria for PreD**	**No. of subjects with PreD**	**Mean follow-up duration (years)**	**Diagnosis of PD**	**No. of subjects who developed PD**	**Variables adjusted**
Saaksjarvi (2015)	Finland	PC	Community-derived population aged 30 to 79 years	6641	50.4	47.1	IFG	1666	30	ICD-10 codes	89	Age, sex, BMI, BP, blood lipids, education, smoking, alcohol consumption, coffee consumption, and serum vitamin D
Rhee (2020)	Korea	RC	People aged 40 years or older for health check-up	15,168,021	55.1	49.1	IFG	2,110,252	3.2	ICD-10 codes	31,577	Age, sex, BMI, smoking status, alcohol consumption, and physical activity
Peng (2021)	China	PC	People aged 60 years or older with mild Parkinsonian signs	1563	66.3	50.2	IFG	623	6	Clinically diagnosed PD	482	Age, sex, BMI, smoking status, alcohol consumption, and prevalence of white matter hyperintensities
Roh (2021)	Korea	RC	Community-derived population aged 40 years or older	171,060	52.5	56.1	IFG	53,228	7.3	ICD-10 codes	819	Age, sex, BMI, income decile, smoking status, alcohol consumption, and physical activity
Sanchez (2021)	Spain	RC	People aged 40 to 80 years attending primary health care centers	2,823,307	68.4	48.2	HbA1c 5.7∼6.4%	266,379	7.3	ICD-10 codes	13,715	Age, sex, BMI, smoking status, and socioeconomic status

PreD: Prediabetes; PD: Parkinson’s disease; PC: Prospective cohort; RC: Retrospective cohort; IFG: Impaired fasting glucose; HbA1c: Hemoglobin A1c; ICD-10: International Classification of Diseases Version 10; BMI: Body mass index; BP: Blood pressure.

**Table 2:** Study quality evaluation using the NOS

**Study**	**Representativeness of the exposed cohort**	**Selection of the non-exposed cohort**	**Ascertainment of exposure**	**Outcome not present at baseline**	**Control for age and sex**	**Control for other confounding factors**	**Assessment of outcome**	**Long enough follow-up duration**	**Adequacy of follow-up of cohorts**	**Total**
Saaksjarvi (2015)	1	1	1	1	1	1	0	1	1	8
Rhee (2020)	1	1	1	1	1	1	0	0	1	7
Peng (2021)	1	1	1	1	1	1	1	0	1	8
Roh (2021)	0	1	1	1	1	1	0	0	1	6
Sanchez (2021)	0	1	1	1	1	1	0	0	1	6

This scale awards up to nine stars, with a higher score indicating a more rigorous study. NOS: Newcastle–Ottawa Scale.

**Figure 2.**
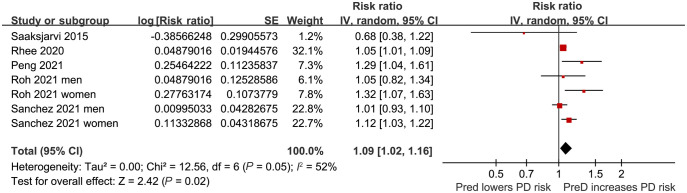
**Forest plots for the meta-analysis of the association between PreD and the risk of PD in the adult population.** PreD: Prediabetes; PD: Parkinson’s disease; SE: Standard error; CI: Confidence interval.

The study excluded reviews, editorials, and meta-analyses. Additionally, studies were excluded if they involved patients with PD at baseline, lacked PreD evaluation, or failed to report on PD incidence outcomes. In instances of overlapping study populations, the meta-analysis incorporated the study with the largest sample size.

### Search of databases

A comprehensive search of electronic databases was conducted, encompassing PubMed, Embase, and Web of Science, spanning from their inception until September 12, 2023. The search aimed to identify studies published within this timeframe. The search strategy involved the combination of the terms: (1) “prediabetes” OR “pre-diabetes” OR “prediabetic” OR “pre-diabetic” OR “prediabetic state” OR “borderline diabetes” OR “impaired fasting glucose” OR “impaired glucose tolerance” OR “IFG” OR “IGT” OR “fasting glucose” OR “HbA1c”; and (2) “Parkinson’s disease” OR “Parkinson disease.” Only studies published in the English language as full-length articles in peer-reviewed journals were included in the analysis. Additionally, a manual screening of relevant original and review articles was conducted to identify potential studies of interest.

### Data extraction and quality evaluation

Two authors independently conducted the literature searches, data collection, and study quality assessments. In the event of discrepancies, a third author was brought in to foster discussion and achieve consensus. Regarding the included studies, exhaustive information was compiled, encompassing study details, characteristics of the study population, participant counts, methodologies employed for diagnosing PreD, follow-up durations, and methods used for confirming PD development in participants during follow-up. Additionally, the analysis recorded variables that were adjusted for assessing the association between PreD and the incidence of PD. The quality of each study was evaluated using the Newcastle–Ottawa Scale (NOS) (19), which assesses studies based on criteria, such as participant selection, group comparability, and outcome validity. This scale awards up to nine stars, with a higher score indicating a more rigorous study.

### Ethical statement

Ethical approval was not required for this study in accordance with local and national guidelines. Likewise, written informed consent for participation in the study was not required, following these guidelines.

### Statistical analysis

In this study, the relationship between PreD and the risk of PD was summarized using RRs and their corresponding 95% confidence intervals (CIs). Whenever feasible, RRs and CIs were extracted from the most comprehensively adjusted regression models. To stabilize variance and normalize the data, RRs and their standard errors (SEs) in each study were transformed logarithmically [28]. The Cochrane *Q* test and the *I*^2^ statistic [29] were utilized to assess heterogeneity among the studies, with an *I*^2^ value over 50% indicating significant heterogeneity. A random-effect model, recommended for accommodating potential inter-study heterogeneity, was used to combine the results [28]. Subgroup analyses were conducted to assess the impact of predefined study characteristics on the outcome. These characteristics included the country of the study, design, age, sex, definition of PreD, and the NOS scores of the included studies. Publication bias was evaluated using a funnel plot and Egger’s regression asymmetry test, which are based on visual symmetry judgments [30]. Statistical analyses were performed using RevMan (Version 5.1; Cochrane Collaboration, Oxford, UK) and Stata software (Version 12.0; Stata Corporation, College Station, TX, USA).

**Figure 3.**
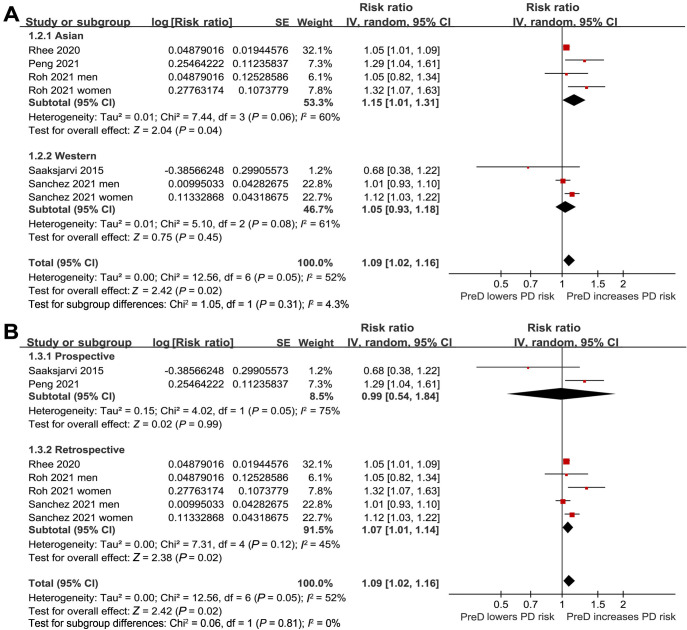
**Forest plots for subgroup analyses of the association between PreD and the risk of PD in the adult population**. (A) Subgroup analysis by the country of the study; (B) Subgroup analysis by the study design. PreD: Prediabetes; PD: Parkinson’s disease; SE: Standard error; CI: Confidence interval.

## Results

### Literature search and study retrieval

Figure 1 depicts the sequential steps involved in the literature search and study retrieval process. The initial database search yielded 212 records. This was followed by the identification and removal of 49 duplicates. A review of titles and abstracts led to the exclusion of 150 studies, as they did not align with the objectives of the meta-analysis. Further scrutiny of the full texts of the remaining 13 studies resulted in the exclusion of additional eight studies, with the specific rationales for exclusion outlined in Figure 1. Consequently, five studies were ultimately selected for inclusion in the final meta-analysis [22–26].

**Figure 4.**
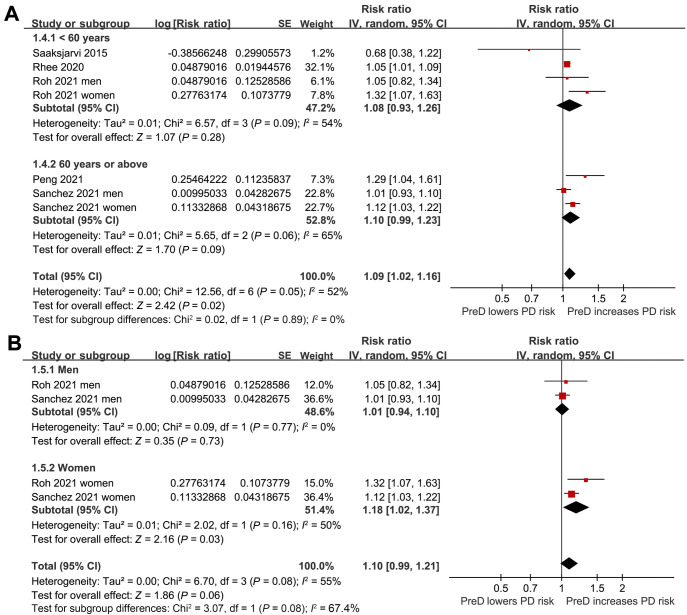
**Forest plots for subgroup analyses of the association between PreD and the risk of PD in the adult population.** (A) Subgroup analysis by the mean age of the participants; (B) Subgroup analysis by the sex of the participants. PreD: Prediabetes; PD: Parkinson’s disease; SE: Standard error; CI: Confidence interval.

### Study characteristics

The meta-analysis encompassed five cohort studies, including two prospective studies [22, 24] and three retrospective studies [23, 25, 26]. The characteristics of these studies are summarized in Table 1. Published between 2015 and 2021, they were conducted in Finland [22], Korea [23, 25], China [24], and Spain [26]. In total, the studies included 18,170,592 adult participants who were not diagnosed with PD at baseline. The mean age of these participants ranged from 50.4 to 68.4 years, with the proportion of male participants varying between 47.1% and 56.1%. PreD was diagnosed based on IFG in four of the studies [22–25] and through mildly elevated HbA1c in one study [26]. Consequently, 2,432,148 participants, or 13.3% of the total, were classified as prediabetic. The mean follow-up durations across these studies varied from 3.2 to 30 years. The PD diagnosis during follow-up was confirmed using the International Classification of Diseases (ICD) codes in four studies [22, 23, 25, 26], while one study [24] utilized clinical diagnosis. A total of 46,682 patients were diagnosed with PD during the follow-up period. The included studies adjusted for potential confounding variables, including age, sex, body mass index (BMI), smoking, and alcohol consumption, to varying degrees. The NOS scores for the included studies ranged from six to eight stars, indicating their moderate to good quality (Table 2).

**Figure 5.**
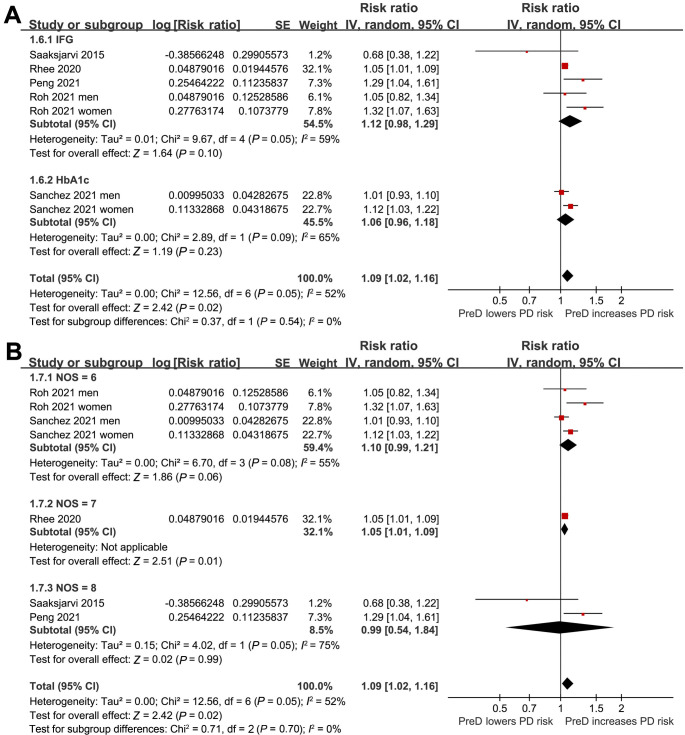
**Forest plots for subgroup analyses of the association between PreD and the risk of PD in the adult population.** (A) Subgroup analysis by the definition of PreD; (B) Subgroup analysis by the quality scores of the studies. PreD: Prediabetes; PD: Parkinson’s disease; SE: Standard error; CI: Confidence interval; IFG: Impaired fasting glucose; HbA1c: Hemoglobin A1c; NOS: Newcastle-Ottawa Scale.

### PreD and the risk of PD in adults

In the meta-analysis, two studies reported results separately for males and females, so these datasets were included independently [25, 26]. Consequently, seven datasets from five cohort studies [22–26] reported the association between PreD and the incidence of PD in the adult population. The pooled results showed that PreD was associated with a higher incidence of PD (RR: 1.09, 95% CI 1.02–1.16; *P* ═ 0.02) (Figure 2), exhibiting moderate heterogeneity (*I*^2^ ═ 52%) after adjusting for potential confounding factors, such as age, sex, BMI, smoking, etc. Subsequent pilot subgroup analyses suggested that the association between PreD and PD might not be significantly affected by the country of the study (*P* for subgroup difference ═ 0.31; Figure 3A), study design (*P* for subgroup difference ═ 0.81; Figure 3B), mean age of the participants (*P* for subgroup difference ═ 0.89; Figure 4A), sex of the participants (*P* for subgroup difference ═ 0.08; Figure 4B), definition of PreD by IFG or mildly increased HbA1c (*P* for subgroup difference ═ 0.54; Figure 5A), or the quality scores of the studies (*P* for subgroup difference ═ 0.70; Figure 5B).

### Publication bias

**Figure 6.**
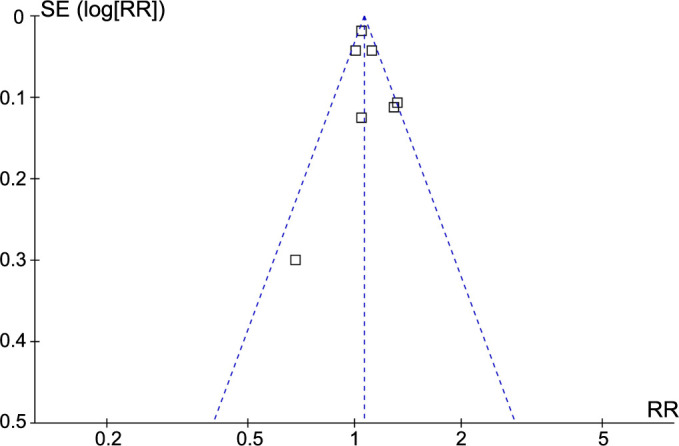
**Funnel plots for assessing publication bias in the meta-analysis of the association between PreD and the risk of PD in the adult population.** PreD: Prediabetes; PD: Parkinson’s disease; SE: Standard error; RR: Risk ratio.

The funnel plots for assessing publication bias in the meta-analysis examining the relationship between PreD and the risk of PD are displayed in Figure 6. Upon visual inspection, the plots exhibit symmetry, indicating minimal publication bias. Furthermore, the application of Egger’s regression tests yielded a low probability of publication bias (*P* ═ 0.33).

## Discussion

This systematic review and meta-analysis synthesized the findings from seven datasets across five cohort studies, demonstrating a potential association between PreD and an increased incidence of PD in the adult population. Subgroup analyses, based on various predefined characteristics, suggested that the association between PreD and an increased risk of PD may not be significantly modified by factors, such as the country of the study, its design, age, and sex of the participants, PreD definition, or the quality scores of the included studies. Overall, the meta-analysis suggests that adults with PreD may face a mildly increased risk of developing PD compared to those with normoglycemia. These findings underscore the potential contribution of glycemic disorders to PD pathogenesis, even at stages preceding diabetes.

The current state of knowledge reveals limited meta-analyses investigating the relationship between PreD and PD. A previous meta-analysis involving 15 cohort studies showed that patients with diabetes have a 27% increased relative risk of developing PD compared to individuals without diabetes [31]. However, this analysis did not explore the association between PreD and PD risk, likely due to the limited datasets involved [31]. In the current meta-analysis, we focused on individuals with PreD, conducting an extensive literature search across three commonly used electronic databases. This search retrieved five up-to-date studies in accordance with the objective of the meta-analysis. By only including cohort studies, our results could potentially indicate a longitudinal relationship between PreD and an increased risk of PD in adults. Multivariate regression analyses were used among all the included studies, and their results suggested that the association between PreD and an increased risk of PD may be independent of factors like age, sex, BMI, and smoking status of the participants. This aspect is important, considering the known correlations of advanced age [32], male sex [33], overweight/obesity [34], and smoking [35] with the risk of PD. The reliability of our findings was further validated through multiple subgroup analyses, indicating that this association might not significantly vary based on predefined study or patient characteristics. Taken together, these results confirmed an association between PreD and an increased risk of PD. However, it is important to note that the association appears relatively weak (RR: 1.09). From our point of view, and from a practical standpoint, routine screening for PD in individuals with PreD is not necessary in general clinical practice. Nonetheless, in high-risk groups, such as older adults, it is advisable to be mindful of an elevated risk of PD in those with PreD compared to those with normoglycemia.

The association between PreD and PD may be multifactorial. Firstly, persistent hyperglycemia, a characteristic of PreD, could subject neurons to chronic metabolic stress, potentially causing subsequent neuronal dysfunction and death, directly leading to PD pathogenesis [21]. Experimental evidence, such as a study on streptozotocin-induced diabetic mice overexpressing human alpha-synuclein, supports this theory. This study observed heightened neuroinflammation and motor dysfunction related to nigrostriatal degeneration, suggesting that hyperglycemia could accelerate PD pathogenesis [36]. Moreover, advanced glycation end-products (AGEs) and hyperglycemia-related glycation agents have also been suggested to participate in the pathogenesis of PD [37]. For example, the receptor for AGEs (RAGE) has been shown to be extensively associated with chronic inflammation in PD, potentially mediating pro-inflammatory signaling in its pathogenesis [38]. Additionally, insulin resistance and associated neuroinflammation have also been involved in the development of PD, possibly through the insulin-like growth factor 1 (IGF-1) signaling pathway. Nevertheless, the precise mechanisms and exact molecular pathways linking hyperglycemia to PD remain to be investigated.

This study is subject to certain limitations. Firstly, the number of available datasets included in this meta-analysis is small. As such, the results, especially those from subgroup analyses, should be interpreted with caution. Secondly, the diagnosis of PreD in the included studies was based on IFG or mildly elevated HbA1c levels. The association between PreD diagnosed with IGT and PD remains to be evaluated in future studies. Additionally, although multivariate analyses were employed in all included studies to observe the association between PreD and PD, the possibility of unadjusted confounding factors that may influence this association cannot be entirely ruled out. Lastly, due to the observational nature of the studies included in this meta-analysis, it is not possible to establish a causal relationship between PreD and an increased risk of PD.

## Conclusion

In conclusion, this meta-analysis indicates that adults with PreD might have a mildly elevated risk of PD compared to those with normoglycemia. This association appears consistent and seems unlikely to be modified by factors, such as the country of the study, its design, the age and sex of participants, the definition of PreD, or the quality scores of the included studies.

## Data Availability

All data generated during this study are included within the manuscript.
